# The effect of hypoxia on hearing function

**DOI:** 10.3906/sag-1902-210

**Published:** 2019-10-24

**Authors:** Nevreste Didem SONBAY YILMAZ, Cem SAKA, Burcu OKTAY ARSLAN, Nurdan AYGENER YEŞİLYURT, Dilek SAKA, Sadık ARDIÇ, İstemihan AKIN

**Affiliations:** 1 Department of Ear, Nose, and Throat, Antalya Education and Training Hospital, Antalya Turkey; 2 Department of Ear, Nose, and Throat, Ankara Dışkapı Yıldırım Beyazıt Education and Training Hospital, Ankara, Turkey; 3 Department of Chest Diseases, Ministry of Health Sciences University,Dr. Suat Seren Chest Diseases and Chest Surgery Training and Research Hospital, İzmir Turkey; 4 Department of Audiology, Antalya Education and Training Hospital, Antalya Turkey; 5 Department of Chest Diseases, Ankara Atatürk Chest Diseases and Chest Surgery Training and Research Hospital, Ankara, Turkey; 6 Department of Chest Diseases, Ankara Koru Hospital, Ankara Turkey

**Keywords:** Chronic obstructive pulmonary disease, auditory brainstem response, distortion-product otoacoustic emission, hearing loss

## Abstract

**Background/aim:**

This study was conducted to determine the critical partial oxygen pressure (pO2) value that would impair hearing function by evaluating the effects of hypoxia on hearing function in subjects diagnosed with chronic obstructive pulmonary disease (CPOD).

**Materials and methods:**

The study included 25 male and 5 female patients referred to our clinic who were diagnosed with COPD, according to spirometry and PaO2 values, and who did not show pathology upon autoscopic examination. The control group consisted of 14 female and 16 male patients who had no lung disease and were in the same age range as the COPD group.

**Results:**

A statistically significant difference was found between the two groups for distortion-product otoacoustic emission (DPOAE) (P < 0.001). The COPD group was divided into two groups according to pO2 levels (pO2 ≤ 70 and pO2 > 70) in order to find a critical pO2 level which might cause significant change at a certain audiological extent.

**Conclusion:**

Hypoxia causes long-term decline in hearing thresholds, deterioration of DPOAE results, and prolongation of I–V interpeak latency in auditory brainstem response. However, the critical oxygen level that disrupts hearing function could not be determined.

## 1. Introduction

Chronic obstructive pulmonary disease (COPD) is a progressive and irreversible systemic lung disease that restricts airflow [1]. Irregular ventilation due to COPD results in hypoxemia, hypercapnia, and, ultimately, hypoxia [2]. These functional abnormalities can be detected by arterial blood gas and pulmonary function tests [1,3]. A decrease in partial oxygen pressure (pO2) is known as hypoxemia, and in practice, it can be observed in any known pulmonary disease [1]. Hypoxia is the consequence of an inability to provide the oxygen required for cell function. Hypoxia in COPD occurs as a result of hypoxemia [4]. 

Functioning of the inner ear depends on the cochlear oxygen supply [3]. Therefore, any decrease in oxygen supply to the cochlea leads to a reduction in cochlear sensitivity [3,5]. It was believed that the reactive oxygen products released during periods of ischaemia and reperfusion cause cochlear damage, and the extent of this damage is due to the length and severity of the ischaemic period [6].

Currently, how hypoxia affects the inner ear is still unknown [7]. Conducting human experiments in artificial hypoxic environments is quite difficult due to both ethical issues and potential complications induced by the systemic effects of hypoxia. Hendricks-Munoz and Walton [8] reported a high risk of progressive hearing loss in infants with persistent pulmonary hypertension, and Hansen [9] suggested that exposure to hypoxia gradually decreases cochlear function in adults. Patients with obstructive sleep apnea were examined, and no difference was observed in their hearing tests. This was attributed to the fact that hypoxia occurs only during sleep, and pO2 was within normal limits in the daytime [7]. Therefore, the study group was altered, and studies have been conducted with patients with consistently low pO2 due to chronic hypoxia-induced COPD. However, testing needs to be done in a quiet environment, and it is extremely difficult to perform audiological tests in patients with COPD due to noisy breathing caused by dyspnea.

In order to predict the impact on hearing function it is important to understand the effects of hypoxia on the inner ear, especially in infants subject to perinatal and postnatal asphyxia. Questions that remain unanswered include: 1) at which level does hypoxia affect hearing pathways (outer or inner hair cells, cochlear nerve, brain stem); 2) which audiological test is the most sensitive test for hypoxia; and 3) is there a critical oxygen value that disrupts hearing function in hypoxia? 

This study was conducted to determine the critical pO2 value that would impair hearing function by evaluating the effects of hypoxia on hearing function in subjects diagnosed with COPD.

## 2. Materials and methods

### 2.1. Patients

The study included 30 patients (25 male and 5 female) diagnosed with COPD, according to spirometry and PaO2 values, ​​and without any abnormalities upon autoscopic examination. The patients were referred to our clinic from the department of chest diseases. The control group consisted of 14 female and 16 male patients with no lung disease and in the same age range as the COPD group.

Exclusion criteria included: 1) ear-related diseases such as acute or chronic otitis media and otosclerosis; 2) history of noise exposure or ototoxic drug use which might affect hearing; 3) a systematic disease other than COPD, such as obstructive sleep apnea, that might cause hypoxia; and 4) a difference of more than 10 dB between two ear hearing thresholds. 

Local ethics committee approval was obtained from S.B. Dışkapı Yıldırım Beyazıt Training and Research Hospital for this study (04.09.2008-04).

### 2.2 Odyological evaluation 

All audiological investigations were carried out in standard double-walled quiet rooms produced by Industrial Acoustics Company (IAC). 

#### 2.2.1. Pure sound audiometry and speech tests 

The audiometric evaluation was performed using Interacoustics AC-40 clinical audiometry. Airway hearing threshold measurements and speech tests were performed using TDH-39 standard headphones, and an Oticon-60273 vibrator was used for bone path threshold measurements. 

The effect of hypoxemia on hearing function is systemic and considered similar for both ears [3]. Therefore, the mean threshold was calculated for each ear at the frequencies tested (250–8000 Hz), and the averages of both ears were taken to obtain a single hearing threshold level (HTL) for each subject.

The speech pick-up thresholds and speech discrimination rates (SD) of the individuals included in the study were also determined.

#### 2.2.2. Tympanogram and acoustic reflex

The middle ear pressure, static impedance, and acoustic reflex thresholds were evaluated using a TDH-39 headset with an Interacoustics AZ-26 impedance meter and a 220 Hz probe tone. The arithmetic means of both middle ear pressures were taken and statistically evaluated.

### 2.3. Electrophysiological evaluation

Measurements were performed in a special, dim test room. The room was protected from the light, and the noise level in the room did not exceed 45 dB.

#### 2.3.1. Distortion-product otoacoustic emission

The distortion-product otoacoustic emissions (DPOAE) (2f1-f2 cubic distortion product components) were measured using the GSI Audera instrument in the general diagnostic mode. The ratio between frequencies f2 and f1 (f2/f1) was fixed at 1.22. The stimulus intensity was L1 for frequency f1 and L2 for frequency f2, and L1-L2 was fixed at 10 dB SPL (L1 = 65, L2 = 55). The test period was about 30 s.

The evaluation of DPOAE results was based on the signal-to-noise ratio in the frequency bands 1, 2, 4, and 8 kHz at the geometric mean of f1 and f2. The averages of these four frequencies were taken for the right and left ears. Statistical analysis was performed with results from both ears.

#### 2.3.2. Auditory brainstem response

Auditory brainstem response (ABR) was measured using the GSI Audera device. The TIPtrode, an extratympanic electrode, was used as the ear canal electrode. Among other disc type electrodes, a positive electrode was placed at the vertex (the top of the head), negative electrodes were placed on the right and left mastoid apex, and the ground electrode was placed on the forehead. During the test, care was taken to ensure that the cables were not overlapping and as far away from the device as possible. In addition, the electrode-skin impedances were kept below 5 kΩ during recording.

Three records were made for each ear; 11.1 click/s at 80 dB hearing level (HL) at adult alternate positive and negative polarity were given as stimulus, and 40 dB of masking was applied to the opposite ear during the test. At least three repetitions were made to reliably determine the result. When more than three repetitions were made, the best overlapping responses were considered for analysis.

The I–V wave latencies and amplitudes and I–V interpeak latencies (IPL) were determined. Then, I–V IPL was recorded separately for both ears, and the mean of both ears were statistically recorded.

### 2.4. Arterial blood gas (ABG) test 

The radial artery was used in all patients for ABG. The pO2 pressure of the patients was noted.

### 2.5. Statistical analysis

Data analysis was performed by SPSS for Windows 11.5. The Shapiro–Wilk test was used to determine whether distribution of continuous variables was normal. Descriptive statistics were expressed as mean ± standard deviation or median (minimum–maximum) for continuous variables, and nominal variables were expressed as number of cases and percent (%). Significance of the difference between groups, in terms of means, was evaluated using Student’s t-test; significance of the difference, in terms of median values, ​​was evaluated using the Mann–Whitney U test. Nominal variables were examined with Pearson’s chi-squared test. The correlation between continuous variables was investigated with Spearman’s correlation test, and P < 0.05 was considered statistically significant.

## 3. Results

A total of 20 male (66.6%) and 10 female (33.3%) patients diagnosed with COPD and with a mean age of 59.4 ± 7.8 years (range: 46–71 years) were included in this study. The control group consisted of 15 male (50%) and 15 female (50%) patients with a mean age of 50.7 ± 9.2 years (range: 41–75 years). The arterial blood oxygen saturation (pO2) of subjects in the control group was 92–100 (mean: 98). In the COPD group, the pO2 was 47–79 (mean: 62). Ten patients with dyspnea and pO2 <60 underwent medical treatment (bronchodilator and nasal oxygen) through chest disease services before starting the test. The HTL, SD, DPOAE, and ABR results of the study groups are shown in Table 1.

**Table 1:** HTL, SD, DPOAE, and ABR results. a. Mann–Whitney U test. B. Student’s t-test.

	Control	CPOD	P-value
HTL	8.3 (2.9–29.5)	31.2 (12.4–51.6)	<0.001a
SD	98.0 (96.0–100.0)	92.0 (60.0–100.0)	<0.001a
DPOAE	17.6 (8.3–27.0)	5.4 (0.2–15.0)	<0.001a
ABR	3.8 ± 0.24	4.0 ± 0.31	0.016b

### 3.1. Hearing threshold level, acoustic reflex, speech discrimination, and tympanogram

In the pure voice audiograms of patients in the control group who did not have any complaints about their hearing, the mean hearing threshold level (HTL) at 250–8000 Hz was 8.3 (2.9–29.5). This value was 31.2 (12.4–51.6) in the COPD group. There was a statistically significant difference between the two groups for HTL (P < 0.001).

Bilateral reflexes were positive in both COPD and control groups, and A type tympanograms were obtained in both groups. 

The mean score of speech discrimination (SD) of both ears was 98 (96–100) in the control group and 92 (60–100) in the COPD group. There was a statistically significant difference between the two groups for SD value (P < 0.001).

### 3.2. DPOAE

The mean of the DPOAE signal-to-noise ratio was 17.6 (8.3–27.0) for the control group. In COPD group, this value was 5.4 (0.2–15.0). A statistically significant difference was found between the two groups for DPOAE (P < 0.001).

### 3.3. ABR

For ABR, I–V IPL was 3.8 ± 0.24 for the control group and 4.0 ± 0.31 for the COPD group. There was a statistically significant difference between the two groups for ABR I–V IPL (P < 0.05).

The COPD group was divided into two groups according to pO2 levels (pO2 ≤ 70 and pO2 > 70) in order to find a critical pO2 level which might cause a significant change at certain audiological extent. There were no statistically significant differences between the HTL, SD, OAE, and ABR results of the 20 patients with pO2 ≤ 70 and 10 patients with pO2 > 70 (P > 0.05) (Table 2).

**Table 2:** According to pO_2_ 70 values HTL, SD, OAE, and ABR results. a. Mann–Whitney U test b. Student’s t-test.

	pO_2_ ≤ 70 (n = 20)	pO_2_ > 70 (n = 10)	P-value
HTL	29.1 (12.4–51.2)	33.9 (19.5–51.6)	0.393^a^
SD	92.0 (60.0–100.0)	93.0 (70.0–94.0)	0.597^a^
OAE	5.2 (0.2–15.0)	5.9 (0.4–13.4)	0.872^a^
ABR	4.0 ± 0.23	4.0 ± 0.49	0.913^b^

The COPD group was divided into two groups: pO2 ≤ 60 and pO2 > 60. There were no statistically significant differences between the HTL, SD, OAE, and ABR test results of the 10 patients with pO2 ≤ 60 and 20 patients with pO2 > 60 (P > 0.05). The 10 patients in group pO2 ≤ 60 could not tolerate the test and received medical treatment beforehand in the chest diseases clinic (Table 3).

**Table 3:** According to pO_2_ 60 values HTL, SD, OAE, and ABR results. a. Mann–Whitney U test b. Student’s t-test.

	pO_2_ ≤ 60 (n = 10)	pO_2_ > 60 (n = 20)	P-value
HTL	29.7 (20.0–51.2)	31.2 (12.4–51.6)	0.475^a^
SD	86.0 (60.0–100.0)	93.0 (70.0–100.0)	0.373^a^
OAE	2.75 (0.2–13.2)	5.75 (0.4–15.0)	0.055^a^
ABR	4.1 ± 0.23	3.9 ± 0.34	0.222^b^

## 4. Discussion

Cochlear oxygenation is important for cochlear sensitivity. When the pO2 pressure decreases, cells respond by reducing their function. The primary cellular damage occurs with reactive oxygen derivatives that are released during reoxygenation. This damage depends on the duration of hypoxia, its occurrence, i.e. suddenly or progressively, and, most importantly, the sensitivity of the cells in the hypoxia region [3,10].

In cases of average oxidative stress, the reactive oxygen products can be compensated for by an increase in the synthesis of the antioxidant defense system. In cases of severe oxidative stress, direct damage to carbohydrates, proteins, lipids, and nucleic acids results in cell death, which renders cochlear damage inevitable. Even when the oxidative stress improves, the cochlear damage is irreversible. Koga et al. [11] initiated an acute severe hypoxia attack in gerbils by obliterating the vertebral artery for 7 days and then developed a reperfusion period by removing the obliteration. Although the blood flow in the vertebral artery was similar to flow during the preischemic period, 10 days after the ischemic attack the loss of the inner shaky hair cells was 6.4% in the basal turn, 6.4% in the second turn, and 0.8% in the apical turn. In addition, an 89% decrease in the spiral ganglion neuron count was reported.

The effect of hypoxia on the cochlea has been attributed to the metabolic alteration of various electrochemical potentials in the ear [12]. These potentials are formed by the metabolic activity of Na/K ions, and it is believed that a decrease in oxygen supply diminishes this process [10]. In chronic hypoxia, hearing loss can be prevented by increasing the number of ion pumps in response to a gradually decreasing oxygen supply [13,14]. Although several studies have investigated the effect of acute hypoxia on hearing, creating a chronic hypoxic environment for animals is very laborious and expensive. COPD is a perfect human model as it creates a hypoxic environment. However, it is extremely difficult to maintain pO2 at a constant level. Therefore, it might not be possible to determine the critical pO2 value leading to hearing loss.

In human studies related to hypoxia, it has been demonstrated that hypoxia decreases hearing thresholds and further reduces SD significantly [3,8,9,15]. In our study, we observed a statistically significant increase in HTL in the COPD group compared to the the control group and a significant decrease in SD compared to the control group. Even in the patient group, there were quite different hearing thresholds compared to the control group. However, in our study, patients with COPD were reluctant to enter the test room due to dyspnea. Anxiety began to occur as test duration increased, and all patients wanted to finish the test and receive oxygen therapy during the test period. Therefore, patient cooperation was low during the determination of pure voice thresholds.

Hypoxia affects otoacoustic emissions as it affects hearing thresholds [12,14,16]. In the first 15 min following cochlear ischemia, swelling and deterioration occurred in the external shaky hair cells. When the external shaky hair cells were examined more closely (ultrastructurally), it was observed that these cells had swelling and distortion in the mitochondria, endoplasmic reticulum, and nuclei [15,17]. Ischemic symptoms caused by acute hypoxia may not improve during the reperfusion period. Morawski et al. [10] found a significant decrease in the internal auditory artery DPOAE level at each frequency, especially at high frequencies, after 5 min of hypoxia followed by a 60-min reperfusion period, compared to the preischemic period. A hypoxic environment in mild hypoxia can be tolerated by increasing the cochlear blood flow. The decrease in DPOAE values in induced hypoxia cases where the pO2 is between 55 and 70 is evident when the oxygen saturation at the time of reperfusion reaches the prehypoxic value, and the resulting free oxygen radicals are held responsible for this [5,16]. Ischemic symptoms cause changes in the basal region of cochlea in less time than that in the apical region.

Sensitivity of the external shaky hair cells to hypoxia causes deterioration in otoacoustic emissions in the early stage of hypoxia. Some researchers have suggested that the cochlear changes caused by hypoxia can be evaluated in the early period by otoacoustic emission measurements [3,10,15,17]. 

However, there are studies suggesting that hypoxia will deteriorate the internal shaky hair cells earlier, and that the first sign of hypoxia is prolongation of I–V waves in ABR before otoacoustic emissions [16].

The most significant effect of hypoxia in ABR is I–V prolongation in IPL [3,17]. A change in ABR waves occurs when the pO2 value reaches the prehypoxic value after reoxygenation, not during hypoxia. Since vasodilatation that is developed during hypoxia increases the cochlear blood flow, it is believed that there is no change in ABR waves during hypoxia [15]. Prolongation of I–V IPL after hypoxia may be due to neuronal stimulation, or it may occur due to the destruction of free oxygen radicals produced according to the severity of hypoxia [18]. However, it is not known whether changes in ABR waves are caused by internal shaky hair cells, nuclei, or the brain stem.

In our study, the ABR I–V IPL value was longer in the COPD group than in the control group, and this difference was statistically significant.

It is well known that hypoxia may cause alterations in hearing thresholds and DPOAE results and prolongation of IPL of I–V waves in IPR in the chronic period [3,4,10]. However, whether there is there a critical level of oxygen that disrupts these values is a matter of debate. El Kady et al. [3] considered pO2 = 70 mmHg as a critical O2 value. In our study, we could not determine a critical oxygen value. We could not detect a statistically significant difference between the hearing thresholds, DPOAE results, and ABR I–V IPL of different patient groups with pO2 >70 and pO2 <70 or pO2 >60 and pO2 <60. However, patients with pO2 <60, in particular, did not want to enter the test room without medical treatment. Therefore, during the test, the pO2 was between 80 and 100. In addition, the test had to be interrupted or the test times were kept shorter as the indoor environment of the test room increased the anxiety of the patients. Therefore, we were not able to determine the critical oxygen value.

In conclusion, COPD is an excellent test environment as it creates chronic hypoxia in humans. Hypoxia causes a decline in hearing thresholds, deterioration of DPOAE results, and prolongation of I–V IPL in ABR over the long term. However, the critical oxygen level that disrupts hearing function could not be determined. There is significant dyspnea and related anxiety in patients with very low pO2. Silence was not achieved in the test room due to noisy breathing, and increased patient anxiety in the closed test room caused the patients to terminate the test. Therefore, it is more appropriate to conduct animal experiments in order to determine the critical oxygen value that will create the effect of hypoxia on cochlea.

## References

[ref0] (2013). Global strategy for the diagnosis, management, and prevention of chronic obstructive pulmonary disease. American Journal of Respiratory and Critical Care Medicine.

[ref1] (2004). The nature of small-airway obstruction in chronic obstructive lung disease. The New England Journal of Medicine.

[ref2] (2006). Study of auditory function in patients with chronic obstructive pulmonary diseases. Hearing Research.

[ref3] (2013). Oxygen therapy in critical illness: precise control of arterial oxygenation and permissive hypoxemia. Critical Care Medicine.

[ref4] (2008). Complex level alterations of the 2f1-f2 distortion product due to hypoxia in the guinea pig. European Archives of Oto-Rhino-Laryngology.

[ref5] (2006). A model of peripherally developing hearing loss and tinnitus based on the role of hypoxia and ischemia. Medical Hypotheses.

[ref6] (2012). Is obstructive sleep apnea syndrome a risk factor for auditory pathway. Sleep and Breathing.

[ref7] (1988). Hearing loss in infants with persistent fetal circulation. Pediatrics.

[ref8] (Supplementum 1988). Postural hypotension–cochleovestibular hypoxia–deafness. Acta Otolaryngological.

[ref9] (2003). Role of mannitol in reducing postischemic changes in distortion-product otoacoustic emissions (DPOAEs): a rabbit model. The Laryngoscope.

[ref10] (2003). Transient cochlear ischemia causes delayed cell death in the organ of Corti: an experimental study in gerbils. The Journal of Comparative Neurology.

[ref11] (2016). Otoacoustic emissions in newborns with mild and moderate perinatal hypoxia. Codas.

[ref12] (1995). Patterns of evoked otoacoustic emissions associated with acoustic neuromas. The Laryngoscope.

[ref13] The effects of chronic hypoxia on human auditory system sensitivity. Aviation, Space, and Environmental Medicine.

[ref14] Estimating the operating point of the cochlear transducer using low-frequency biased distortion products. The Journal of the Acoustical Society of America 2009 Apr; 125.

[ref15] (2003). Preventing internal auditory artery vasospasm using topical papaverine: an animal study. Otology &amp; Neurotology.

[ref16] (2014). Complex level alterations of the 2f(1)-f(2) distortion product due to hypoxia. Auris Nasus Larynx.

[ref17] (1994). Effects of hypobaric hypoxia on the human auditory brainstem responses. Hearing Research.

